# Use of Micronization and Complex Coacervation to Preserve Antioxidant Properties of Flavonoids

**DOI:** 10.1155/2023/9456931

**Published:** 2023-09-15

**Authors:** Rinat Fatkullin, Irina Kalinina, Natalya Naumenko, Ekaterina Naumenko

**Affiliations:** Department of Food and Biotechnology, South Ural State University (National Research University), 76 Lenin Avenue, Chelyabinsk 454080, Russia

## Abstract

The plant flavonoids taxifolin and rutin are among the best known and best studied antioxidants. In addition to their antioxidant properties, other pharmacobiological properties have been established for these substances. At the same time, taxifolin and rutin are chemically labile. They are prone to oxidative degradation and have poor water solubility. Under conditions of their real consumption, all this can lead to a significant reduction or complete loss of bioactivity of these flavonoids. Flavonoid modification and encapsulation techniques can be used to overcome these barrier factors. The use of micronization process for taxifolin and rutin allows changing the lipophilicity values of antioxidants. For micronized taxifolin, the log *P* value is 1.3 (1.12 for the control forms), and for rutin, it was 0.15 (-0.64 for the control forms). The antioxidant activity of micronized flavonoids has increased about 1.16 times compared to control forms. The present study evaluates the possibility of using encapsulation of premyconized flavonoids by complex coacervation, in order to preserve their antioxidant properties. The results of an in vitro digestion study show that the encapsulated forms of antioxidants retain their bioactivity and bioavailability better than their original forms. The bioavailability indices for the encapsulated forms of flavonoids are more than 1.6 times higher than for their original forms. The digested fractions of the encapsulated properties reveal better antioxidant properties than their original forms in in vitro tests evaluating the antioxidant properties on cultures of the protozoan *Paramecium caudatum* and human neuroblastoma SH-SY5Y cells. Encapsulated rutin indicates the highest activity, 0.64 relative to PMA. Thus, the studies represent the feasibility of using encapsulation to protect flavonoids during digestion and ensure the preservation of their antioxidant properties.

## 1. Introduction

Plant-based biologically active substances (BAS) have shown numerous pharmacological effects on human health in clinical studies. Many of them can be used for the nonmedicinal correction of noninfectious diseases, such as cardiovascular and metabolic diseases, as well as some forms of cancer [1–3]. Many studies show the enormous role of polyphenols and flavonoids in minimizing the risks of the negative effects of oxidative stress and the likelihood of hypertension, diabetes, cancer, and other noncommunicable diseases [4–8].

Taxifolin and rutin are unique bioactive flavonoids, dietary components that have captured the interest of dietitians and medicinal chemists due to the wide range of health benefits. These are powerful antioxidants with a well-documented effects in the prevention of several malignancies in humans. Taxifolin has shown promising inhibitory activity against inflammation, malignancies, microbial infection, oxidative stress, cardiovascular disease, and liver disease. Rutin also has several pharmacological activities including antiallergic, anti-inflammatory and vasoactive, antitumor, antibacterial, antiviral, and antiprotozoal properties [9–13]. For this reason, the use of these plant bioactives is gaining in interest due to their prescribed role in human health research [5, 8, 9].

The use of micronization process for taxifolin and rutin allowed us to change the lipophilicity values of antioxidants. For micronized taxifolin, the log *P* value was 1.3 (1.12 for the control forms), and for rutin, it was 0.15 (-0.64 for the control forms). The antioxidant activity of micronized flavonoids has increased about 1.16 times compared to control forms [12].

Bioavailability of biologically active substances largely depends on their solubility and ability to withstand digestion. Therefore, poorly soluble and chemically unstable substances, such as flavonoids, demand a search for technological approaches to overcome these problems. One of such solutions may be micronization, which reduces the particle size of the substance, thus potentially increasing its solubility and the degree of penetration through cell membranes. The use of encapsulation can provide more efficient delivery of biologically active substances through the digestive system, protecting them from degradation [14–16].

The advantages of micronization approaches to improve the bioavailability and bioactivity of flavonoids have been confirmed by numerous studies. The micronized biologically active substances are therapeutically effective due to their significant size reduction. These substances are easily dissolved and absorbed in gastrointestinal tract, and then, they get into the blood stream. Traditionally micronization methods have been used in the pharmaceutical industry, but in recent decades, various studies have revealed their great potential in the food industry [17, 18, 19].

However, micronization technologies do not resolve the problem of low chemical stability of taxifolin and rutin, their tendency to degradation under the influence of external influences, and the possible loss of biological activity due to stable interactions with macromolecules of the food matrix [16, 18, 20].

For this reason, new technological solutions need to be developed to minimize the quantitative losses of biologically active compounds and to ensure the required level of biologically active compounds' technological suitability, bioavailability, and bioactivity [5, 21, 22].

One such solution could be encapsulation technologies, among which microencapsulation and nanoencapsulation [23, 24].

Encapsulation technologies, including microencapsulation and nanoencapsulation, are common in the food and pharmaceutical industries. They increase the effectiveness of polyphenols, trace elements, enzymes, etc. These technologies reduce the degradation of biologically active substances under the influence of external factors, such as pH, temperature, light, oxygen, and mechanical influences. Encapsulation involves protecting of BAS a carrier material when particles or capsules are formed on the micrometer or nanometer scale [25, 26].

The main objective of BAS encapsulation in the creation of healthy foods is to increase their bioavailability and preserve pharmacological properties by protecting the BAS from external negative influences in the technological process of production of the product and at its use [27, 28].

The trigger factor determining the success of the process of encapsulation of biologically active substances is the choice of the carrier material.

At the same time, it is important to consider certain characteristics for selecting the agents for encapsulation:

- In terms of safety, encapsulating agents must be approved as biodegradable and safe (in EU countries, “generally recognized as safe substances” are labeled GRAS) materials for food
- Encapsulating agents should effectively protect biologically active compounds from the negative effects of external stress factors
- They must have acceptable processing properties, which are generally low viscosity at high concentration, good emulsifying capacity, and solubility [29, 30]

It is ideal if the encapsulating substances are able to provide “targeted delivery” by overcoming the acidic and enzymatic state of the gastrointestinal tract, as well as increase the penetrating ability of BAS.

The most widely used resources for BAS encapsulation are natural biopolymers, among which are proteins, carbohydrates, and fats. As a rule, they are biodegradable and nontoxic for the human body. Polysaccharides such as starch, cyclodextrins, and pectins are widely used among carbohydrates and among proteins—gelatin, zein, gliadin, and whey proteins. Lipids are most applicable in emulsion technology, for which vegetable oils and various surfactants are used [31–33].

**Figure 1:**
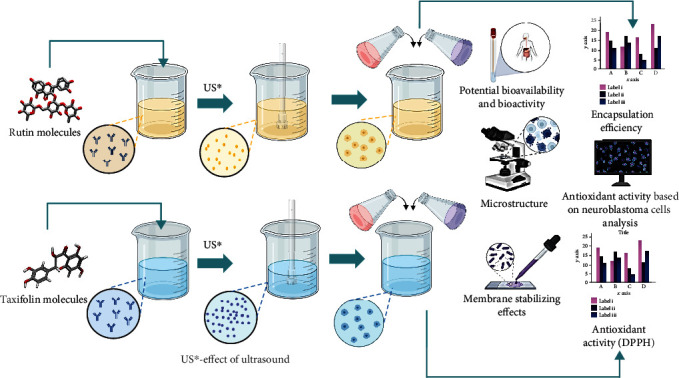
Generalized scheme of research.

The aim of the present study was to develop encapsulated flavonoid complexes with preliminary micronization and to evaluate the effect of this approach on the preservation of bioactive properties of antioxidants (Figure 1). Complex coacervation was chosen as the encapsulation method. Despite the wide presentation of studies on complex coacervation in the literature, the combination of micronization and encapsulation is new and has not been studied sufficiently.

## 2. Materials and Methods

### 2.1. Subjects of Research

In this study, the flavonoids rutin and taxifolin were chosen as BAS. They are the most studied plant antioxidants, promising for use as food ingredients in the production of functional, fortified, and specialized food products.

**Figure 2:**
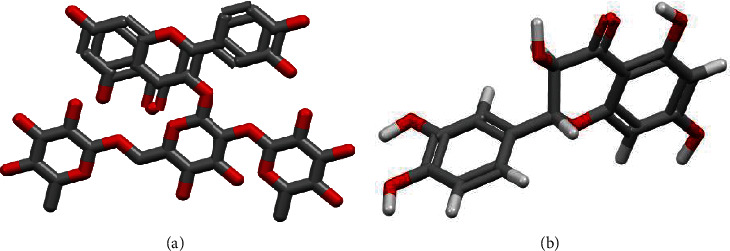
Spatial structure of rutin molecules (a) and taxifolin (b) [34].

Their general characteristics and molecular structures are shown in Figure 2 and Table 1, respectively.

The objects of the study were defined powdered forms of flavonoids purchased in the trade network of Chelyabinsk:

- Taxifolin, manufacturer LLC “Taxifolia” (Belgorod, Russia). Certificate of state registration number RU 77.99.003 E.018404.05.11 from May 6, 2011. Purity of 98-99%
- Rutin, manufacturer NOW Foods (New York, State of New York, USA); the source—flowers Sophora japonica

Excipients for encapsulation were used:

- Beef gelatin, manufacturer Dr. Oetker (Bielefeld, Germany), purchased in the retail network of Chelyabinsk
- Citrus pectin, manufacturer Valde (Moscow, Russia), purchased in the trade network of Chelyabinsk

### 2.2. Micronization Conditions

Micronization of flavonoids was carried out using the effects of ultrasound (US). The ultrasonic device “VOLNA-L” UZTA-0,63/22-OL (Biysk, Russia) with a fungus-type working tool was used as a working tool. Initial flavonoid solution of 0.2% in the volume of 100 ml was treated using the working mode of exposure: 630 W, 5 min at a temperature control of not more than 50°C, as described in detail in [35–39]. Micronized flavonoids were lyophilically dried.

Next, the encapsulation process was performed as described in Section 2.3.

### 2.3. Encapsulation Conditions

The encapsulation of flavonoids was carried out according to the method [40]. Briefly, 50 milliliters of 2% *w*/*v* of pectin and gelatin solutions was prepared separately by heating at 45°C. The pH of each solution was adjusted to 7.5 using 1 N sodium hydroxide before making up the volume to 50 ml. Flavonoids in a ratio of 1 : 1 by weight to gelatin were introduced into the gelatin solution by magnetic stirring for 15 min until a clear solution or suspension without lumps was obtained. Then, 50 ml of pectin solution was added to gelatin solution and stirred well using magnetic pellet (approx 500 rpm). The temperature of the mixture was maintained at 45°C, and pH was reduced from 7.5 to 5 by fast addition of 0.5 N HCl. Then, the 0.5 N HCl was added by drops, in order to bring down the pH to 3.8 to induce coacervation. The precipitated microcapsules were frozen in liquid nitrogen, lyophilized for 48 h, and stored in a desiccator until further analysis.

The objects of the study were defined as follows:

- Taxifolin control (Tcontr)—taxifolin in its native form
- Rutin control (Rcontr)—rutin in its native form
- Taxifolin encapsulated (Tencaps)—a 0.2 ± 0.001 g sample of taxifolin was dissolved in 100 ml of distilled H_2_O, subjected to ultrasound at 20 ± 2 kHz and 630 W, for 5 min with a temperature control not exceeding 50°C
- Rutin encapsulated (Rencaps)—rutin sample weighing 0.2 ± 0.001 g was dissolved in 100 ml of distilled H_2_O, subjected to ultrasound at 20 ± 2 kHz and 630 W for 5 min at a temperature control of not more than 50°C

### 2.4. Nomenclature of Indicators and Methods of Analysis

The samples of encapsulated taxifolin and rutin were evaluated in comparison with the original species, according to the following indicators using the methods described.

#### 2.4.1. Solubility

The thermodynamic solubility of the selected compounds in water was investigated using the following approach: an excess of each substance was added to 5 ml of distilled water and stirred in a thermostatically controlled shaker while maintaining a speed of 200 rpm for 60 min. The supernatant was filtered by a membrane filter (0.45) after centrifuge process (5000 rpm, 5 min). In the filtrate obtained, the content of the active substance was determined spectrophotometrically, using a Jenway spectrophotometer (6405 UV/Vis, UK), using aluminum chloride (Komponent Reagent LLC, Moscow, Russian Federation) spectrophotometrically by the method of State Pharmacopoeia XIV. Experiments were performed at several temperatures and time points [41]: 20, 35, and 50°C and 20, 40, and 60 min.

#### 2.4.2. Lipophilicity

Lipophilicity (log *P*) was established by determining the logarithm of the ratio of BAS concentrations in the system of two immiscible liquids: 1-octanol and water.

Calculation of log *P* is done by the following formula:

(1) $$\begin{array}{c} \log\;P=\log\;\frac{C_{\text{octanol}}}{C_{\text{water}}}, \end{array}$$

where *С*_octanol_ is the concentration of the dissolved substance in 1-octanol (mg/ml) and *С*_water_ is the concentration of the dissolved substance in water (mg/ml).

#### 2.4.3. The Morphological Structure

The morphology of the encapsulated complexes was studied by preparing uncolored crushed drop preparations using transmission microscopy (microscope Micromed S-11, Russia) at magnification *×*640.

#### 2.4.4. The Total Antioxidant Activity (AOA)

Total antioxidant (antiradical) activity was determined by DPPH method (%) [42, 43].

A methanol solution of DPPH (60 microns) was mixed with the test solution in a ratio of 1 : 1 and kept for 30 minutes without light access. Absorption was measured using a Jenway spectrophotometer (6405 UV/Vis, UK) with wavelength of 515 nm.

The AOA was calculated using the following formula:

(2) $$\begin{array}{c} \text{AOA}=\frac{1-\left(D_{\text{i}}-D_{\text{j}}\right)}{D_{\text{c}}}\times 100, \end{array}$$

where *D*_i_ is the optical density of the test solution, *D*_j_ is the optical density of DPPH control solution with methanol, and *D*_c_ is the optical density of DPPH solution.

#### 2.4.5. Total Flavonoid Content

The quantitative content of flavonoids was determined by interaction with aluminum chloride spectrophotometrically by the method of State Pharmacopoeia XIV, described in more detail in [20].

#### 2.4.6. Encapsulation Efficiency (EE)

To evaluate the effectiveness of the process of loading flavonoids into coacervates, the Sun method was used [37]. The encapsulation efficiency was determined taking into account the amount of flavonoids encapsulated and remaining on the surface of the capsules. The calculation was carried out according to the following formula:

(3) $$\begin{array}{c} \text{EE }\left(\%\right)=\frac{C1-C0}{C2}\times 100, \end{array}$$

where *C*1 is the total amount of bioactive substance after the destruction of capsules (mg), *C*2 is the amount of nonencapsulated bioactive substance (mg), and *C*0 is the initial amount of bioactive substance (mg).

#### 2.4.7. Potential Bioavailability and Bioactivity

Potential bioavailability and bioactivity are based on the determination of the bioactivity index (*I*_ba_) and bioavailability index (*I*_bav_) by the method [44, 45].

In vitro digestion simulation included 3 stages:

*Cleavage in the oral cavity*: alpha-amylase enzyme in saline solution, pH 7, temperature 37° C, and 15 min

*Cleavage in the stomach*: pork pepsin, pH 2.5 (using HCl), temperature 37°C, and 2 hours in the dark

*Cleavage in the intestine*: pancreatin and lipase enzymes, pH 6.5-7, temperature 37°C, and 2 hours in the dark

The resulting mixture was centrifuged and filtered through a membrane (0.45 microns).

The content of each of the flavonoids and AOA (DPH, %) is determined in the filtrate.

The bioactivity index (*I*_ba_, %) is calculated by the following formula:

(4) $$\begin{array}{c} I_{\text{ba}}=\frac{АО{А}_{\text{conc}}}{\text{AO}{А}_{\text{orig}}}\times 100, \end{array}$$

where AOA_conc_ is the AOA after the digestion process and AOA_orig_ is the AOA before the digestion process.

Bioavailability index (*I*_bav_, %) is calculated by the following formula:

(5) $$\begin{array}{c} I_{\text{bav}}=\frac{K_{\text{conc}}}{K_{\text{orig}}}\times 100, \end{array}$$

where *K*_conc_ is the amount of flavonoid after the digestion process and *K*_orig_ is the amount of flavonoid before the digestion process.

#### 2.4.8. Analysis of Bioavailability and Membrane-Stabilizing Effects

The antioxidant and membrane-stabilizing effects of flavonoids were determined according to the method proposed by Stepanova et al. [46]. According to this technique, a culture of protozoa, Paramecium caudatum infusoria, was used as a test system. Ethyl alcohol was used as poisons in the study of membrane-stabilizing activity, and hydrogen peroxide solution was used for antioxidant activity. The infusoria were counted on a BioLaT-3.2 device using the AutoCiliataXP program [47].

#### 2.4.9. Assessment of Antioxidant Activity by the Intensity of Reactive Oxygen Species (ROS)

We used a cell model of induced generation of reactive oxygen species in a culture of undifferentiated human neuroblastoma SH-SY5Y cells. The cell line was obtained from the Institute of Cognitive Neuroscience (London, UK) and deposited in the genetic bank of the Institute of Cell Biophysics, Russian Academy of Sciences.

The cells were cultured in DMEM supplemented with 10% fetal bovine serum.

Cells were grown on culture mats (25 cm^2^) until 80% confluence. Cultivation was performed in a humidity-controlled CO_2_ incubator at 37°C and a CO_2_ content of 5%.

To assess the intensity of active oxygen species generation in neuroblastoma cells, we used the method described in [46].

Fluorescent dye 2,7-dichlorodihydrofluorescein diacetate (DCFH2-DA) was used as a control and in the presence of the studied substances. The dye was loaded into the cells for 30 min at 37°C. The dye concentration was 10 *μ*m. Incubation with samples was performed for 2 hours, after which cells were washed (dye was added 30 min before washing). Then, phorbol ester (phorbol-12-myristate-13-acetate (PMA)) was added to the cell culture to induce the generation of reactive oxygen species. All experiments were performed in HBSS (Hank's balanced salt solution) medium with the addition of HEPES to maintain buffer capacity.

Fluorescence was recorded using a Tecan Spark 10 M fluorescence plate reader with a thermostatically controlled camera. To visualize cell cultures using fluorescence microscopy, we used a system based on a Leica DMI6000B inverted motorized fluorescence microscope, with a HAMMAMATSU C9100 monochrome CCD camera, and Leica EL6000 light source with a HBO 103 W/2 mercury high pressure lamp. A fluorescence cube A (Leica, Germany) with excitation filter BP 340-380, dichroic mirror 400, and emission filter LP 425 was used to excite Hoechst 33342 fluorescence. The images were taken using a Leica HCX PL APO lambda blue 63.0 × 1.40 oil lens. To be able to compare the dye fluorescence intensities in the cultures incubated with the samples, the imaging was performed with the same settings that were used to photograph the control sample.

#### 2.4.10. Statistical Processing of the Results

When obtaining and processing the results, fivefold repetition of the studies was observed. All micronization and encapsulation processes were performed under the same conditions. Processing of the results of the studies was carried out using methods of mathematical statistics (Microsoft Excel and Mathcad). All results were presented as mean value ± standard deviation. A *P* value < 0.05 was considered a statistically significant difference. All studies were performed by accredited laboratories in accordance with GOST R ISO 5725-2-2002.

## 3. Results

### 3.1. The Study of the Properties of the Initial Forms of Taxifolin and Rutin

**Figure 3:**
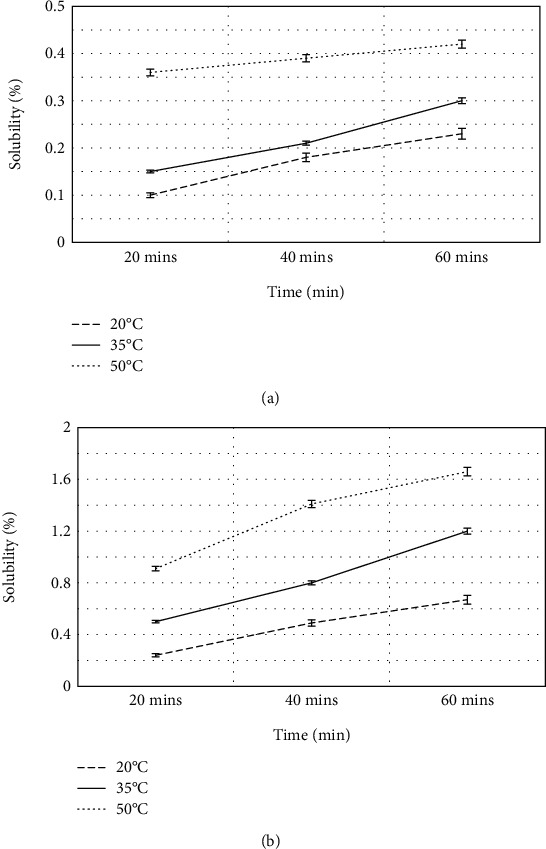
Dissolution rate profile of flavonoid control samples: (а) Tcontr; (b) Rcontr. The error bars represent the standard deviation of measurements (*n* = 5).

The data obtained show a fundamental difference in the solubility of the studied flavonoids (Figure 3). The closest values of solubility in water were characterized by taxifolin and rutin at 20°C. After 20 min, the value was about 0.06-0.08%. This indicates an extreme degree of hydrophobicity of these compounds. The duration of the dissolution process for flavonoids did not make significant adjustments at this temperature.

Increase in temperature led to almost 2-fold increase in solubility of rutin in water. However, the actual values of the transition of the substance in solution remained low.

By analyzing the maximum solubility values of taxifolin and rutin at the maximum dissolution point (50°C and 60 min), it can be noted that the solubility of taxifolin is significantly inferior to that of rutin (more than 4 times).

**Figure 4:**
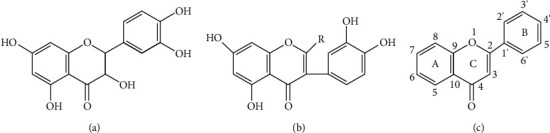
Structural formula of taxifolin molecules (a), rutin (b), and the general scheme of the image of polyphenol molecules (с).

When comparing the structural features of the molecules of the studied substances, we see that the structure of rutin and taxifolin molecules is akin (Figure 4). At the same time, we can note the different positions of -OH substituents in the B ring in contrast to taxifolin—these are positions 3 and 4, as well as the presence of a double bond in positions 2-3 of the C ring [11, 13, 48]. According to [49, 50], the C2-C3 double bond as well as the connection of the B ring with the C ring through the C3 bond significantly increases the solubility of flavonoids in water, as confirmed by our data. However, despite the higher solubility of rutin in water compared with taxifolin, the solubility values for this substance did not exceed 2%.

Absorption of bioactive substances accompanying food or dietary supplements is mainly determined not only by their solubility but also by their permeability through cell membranes. These substances must be soluble in the aqueous environment of the gastrointestinal tract and be able to penetrate through the mucous membrane. In this case, they will be able to enter the systemic bloodstream and exhibit their pharmacological activity.

The results obtained in this study are presented in Table 2.

When comparing the data on the assessment of the lipophilicity of the studied flavonoid samples, we can note a very high variability of values. The range was from -0.64 to 1.12. In this case, the most unacceptable values, close to the limits, are noted for rutin.

Taken together, the obtained results of solubility and lipophilicity indicate the need to find ways to correct the properties of the studied flavonoids and increase their bioavailability and antioxidant properties. This is possible through the use of microstructuring and encapsulation.

### 3.2. Effect of Micronization on Flavonoid Properties

A study of the solubility of micronized taxifolin and rutin under conditions corresponding to the maximum solubility values of their original forms (50°C, 60 min) showed an increase in values by more than 1.5 times for taxifolin and by 1.7 times for rutin. The results agree with the studies [11, 50, 51] and with the results obtained earlier by our group. The increase of solubility values agrees with the Neuss-Whitney dissolution theory, which is probably caused by a decrease in flavonoid particle size due to ultrasound exposure [50, 52, 53] and by an increase in the solid-solvent interaction area.

The values of flavonoid lipophilicity also changed. For micronized taxifolin, the log *P* value was 1.3, and for rutin, it was 0.15. We consider the lipophilicity index as a prognostic estimate of flavonoid bioavailability. Even though this model predominantly characterizes hydrophobic interactions, we can say that micronization gives a positive shift in the values of this index for both taxifolin and rutin.

**Figure 5:**
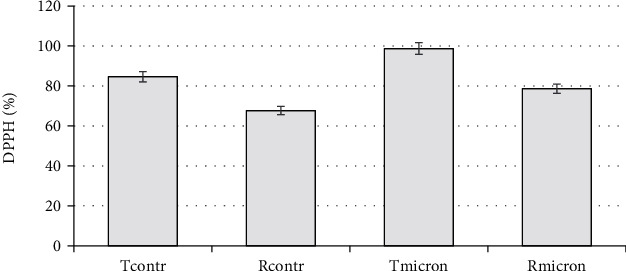
AOA (DPPH, %) of aqueous solutions (0.01%) of initial and micronized. The error bars represent the standard deviation of measurements (*n* = 5).

The evaluation of the antioxidant activity of micronized flavonoids (Figure 5) shows a positive effect of micronization on the ability to bind free radicals in the DPPH method model. The results presented reflect an increase in the values of antioxidant activity. This agrees with the results of studies by other authors [54] and may be related both to an increase in the solubility of the substances and to an increase in the number of hydroxyl groups because of the depolymerization process. Certain studies have reported that the leading role in the formation of the antioxidant properties of flavonoids belongs to hydroxyl groups [13].

### 3.3. Effect of Encapsulation on Flavonoid Properties

**Figure 6:**
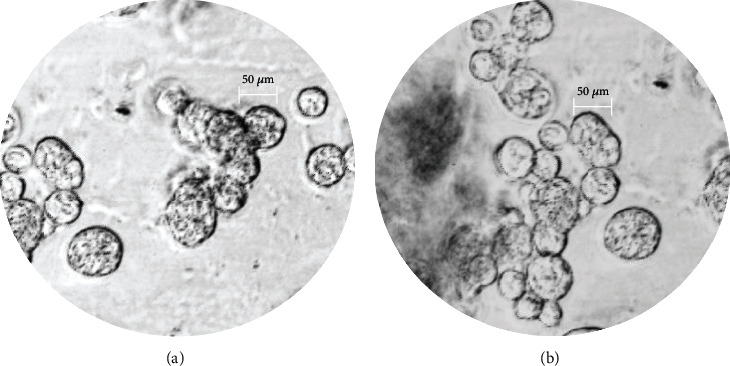
Results of flavonoid microscopy (preparation-crushed drop, magnification ×640): (а) Tencaps; (b) Rencaps.

The results of the study of the morphological characteristics of the encapsulated complexes of taxifolin (Figure 6(a)) and rutin (Figure 6(b)) show the spherical nature of the complexes obtained. The sizes of the capsules are quite one normal and are in the range of 30-50 *μ*m. Studies [55] showed that the morphology of the empty gelatin-pectin coacervate was spherical and translucent. Microscopic images of the coacervates we obtained clearly demonstrate multinucleated spheres. This shows that flavonoids were surrounded by gelatin and pectin. Consequently, we assumed that the biologically active substances were successfully encapsulated.

According to the data available in the literature, the encapsulation efficiency when using complex coacervation is on average 60-80% and depends to a large extent on the correctly chosen conditions for the encapsulation process, especially the pH values.

**Figure 7:**
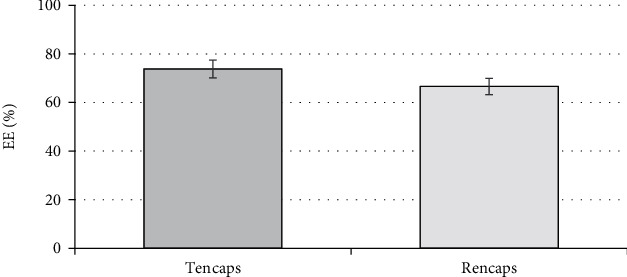
The effectiveness of the proposed method of encapsulation of flavonoids. The error bars represent the standard deviation of measurements (*n* = 5).

The results of the efficiency determination are presented in Figure 7.

The results showed that the efficacy values of the proposed encapsulation method using both taxifolin and rutin have high values (73.8% and 66.6%, respectively).

At the same time, to assess the feasibility and efficiency of the proposed modification and encapsulation technologies, it is necessary to evaluate their effect on the preservation of bioactivity of flavonoids. Therefore, we attempted to study the role of encapsulation technologies with premicronization for saving the antioxidant properties of flavonoids during their digestion.

The studies showed that the encapsulation process leads to a decrease in the values of the flavonoids' antioxidant activity: for taxifolin, by 33.9%, and for rutin, by 52.1%.

These results can be explained by the fact that the complex encapsulating coating of gelatin and pectin covers the biologically active substance, shielding the functional groups of flavonoid molecules, including hydroxyl ones that participate in the formation of the antioxidant effect [41]. At the same time, in order to evaluate the efficiency of the selected encapsulation technologies, their effect on the biological activity of flavonoids needs to be evaluated. Therefore, the present study has attempted to investigate the role of encapsulation technologies with premicronization for preserving the antioxidant properties of flavonoids during their digestion.

**Figure 8:**
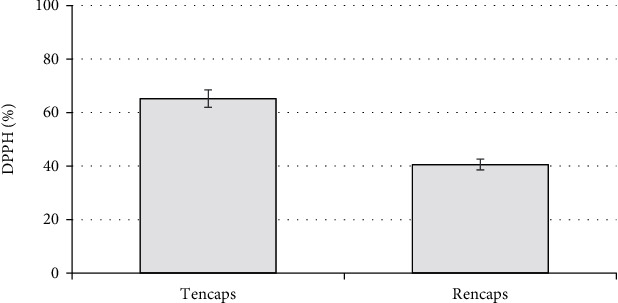
AOA (DPPH, %) of aqueous solutions (0.01%) of initial and encapsulated flavonoids. The error bars represent the standard deviation of measurements (*n* = 5).

The studies have showed that the encapsulation process affects the antioxidant properties of flavonoids (Figure 8).

**Figure 9:**
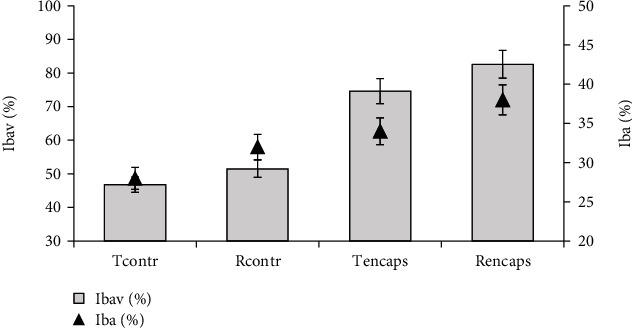
Potential bioactivity (*I*_ba_) and bioavailability (*I*_bav_) of aqueous solutions (0.01%) of starting and encapsulated flavonoids. The error bars represent the standard deviation of measurements (*n* = 5).

The results of determining the bioavailability and bioactivity indices of encapsulated flavonoids in comparison with their original forms are shown in Figure 9.

Many studies have confirmed that encapsulation technologies can minimize the degradation processes of biologically active substances and thus increase their bioavailability in the human body. For example, the bioavailability of icaritin increased by 28 times when it is encapsulated in pectin nanoparticles [56]. The bioavailability of quercetin increased when it is encapsulated in casein nanoparticles as well as zein nanoparticles [57, 58].

Encapsulation of beta-carotene was able to increase its bioavailability during in vitro digestion by about 6-fold [56].

The positive effect of other protein carriers on the bioavailability of some lipophilic compounds has been shown in Grace et al. [59].

Our studies have also revealed the feasibility of using complex coacervation to reduce the quantitative loss of flavonoids during digestion. The bioavailability of encapsulated taxifolin and rutin increased approximately by 1.56-fold compared to their original forms.

At the same time, it is important to track not only the quantitative preservation of biologically active substances but also the preservation of their biological activity. In our case, we monitored the antioxidant properties.

**Figure 10:**
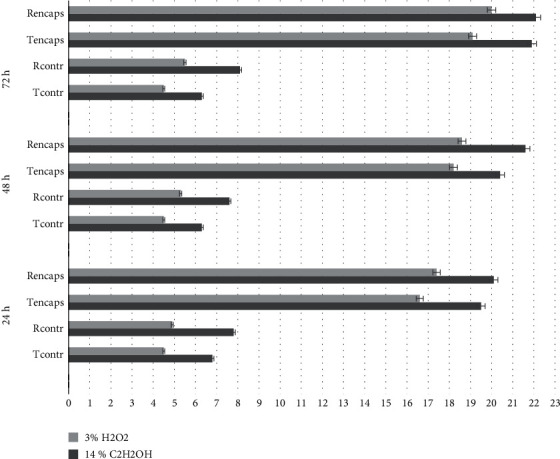
Antioxidant and membrane-stabilizing effects of aqueous solutions (0.01%) of the initial and encapsulated flavonoids. The error bars represent the standard deviation of measurements (*n* = 5).

The results of determining the antioxidant and membrane-stabilizing effects of the initial flavonoids and their encapsulated complexes using *Paramecium caudatum* infusoria are shown in Figure 10.

Based on the data obtained and in accordance with the criteria for evaluating the antioxidant and membrane-stabilizing activity, the flavonoid samples (Tcontr and Rcontr) can be classified as moderately active substances (by the infusorium stopping time and by the lysing poison concentration). The encapsulated flavonoid samples (Tencaps and Rencaps) have a high level of activity for the above two characteristics, which may indirectly indicate high antioxidant properties [47–49, 60, 61].

**Figure 11:**
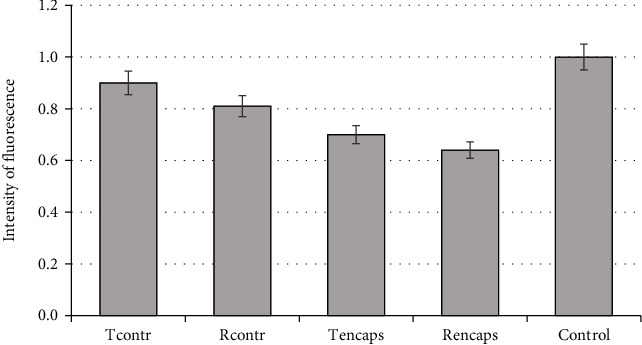
Intensity of ROS generation after 3 h (in the control with PMA taken as a unit, all other values are given relative to the control). The error bars represent the standard deviation of measurements (*n* = 5).

The results of measuring the inhibitory properties of flavonoids against the formed ROS in the neuroblastoma cell culture model are shown in Figure 11.

The data presented in the figures demonstrate that Rencaps is the most active in blocking ROS (0.64 versus PMA control). The Tencaps sample was inferior to the results (fluorescence intensity 0.7 relative to PMA control). The initial forms of flavonoids, on the other hand, exhibited antioxidant properties more than 20% lower than the encapsulated forms.

## 4. Conclusions

Thus, the presented materials and studies have shown the expediency of using an integrated approach to obtain more effective forms of taxifolin and rutin, based on the preliminary micronization of substances and their subsequent encapsulation by coacervation in gelatin and pectin.

Such technology increases bioavailability of plant antioxidants under conditions of in vitro digestion more than 1.5 times with a slight increase in their bioactivity.

In addition, the evaluation of antioxidant and membrane-stabilizing effects of flavonoids in the model of cell cultures and protozoa demonstrated a significantly higher level of activity of encapsulated forms of flavonoids.

The proposed technology can be used in obtaining effective dietary supplements and food ingredients for nonmedicamentous and preventive correction of noninfectious diseases.

The effectiveness of the considered approaches for obtaining food ingredients can be evaluated by additional studies, involving the behavior evaluating of encapsulated forms of flavonoids in the composition of food systems.

## Figures and Tables

**Table 1:** Chemical characteristics and physical properties of flavonoids [34].

Property	Value	
	Rutin	Taxifolin
Solubility in water	12.8 g/l	1.16 g/l
log *P*	-0.54	1.07
Physiological charge	-1	0
Count of hydrogen acceptors	21	7
Count of hydrogen donors	13	5
Polar surface area	344.67 Å^2^	127.45 Å^2^
Refraction	172.56 m^3^·8 mole^−1^	74.61 m^3^·mole^−1^
Polarizability	73.13 Å^3^	29.03 Å^3^
Number of benzoic rings	6	3
Molecular mass	610.52	304.3

**Table 2:** Lipophilicity of the flavonoids under study.

Property	Meaning	
	Tcontr	Rcontr
Lipophilicity (log *P*)	1.12 ± 0.03	−0.64 ± 0.02

## Data Availability

Data will be made available on request to the corresponding author.
